# Supplementary material for: Pendred Syndrome, or Not Pendred Syndrome? That Is the Question
Source: Genes (Basel). 2021 Oct 1;12(10):1569. doi: 10.3390/genes12101569 (PMC8535891; doi:10.3390/genes12101569)
Supplement: Supplementary file 1 [file genes-12-01569-s001.zip › genes-1392592-supplementary.pdf]

Table S1: Patients' hearing thresholds. Hearing thresholds (expressed in dB) at different frequencies (expressed in Hertz) of the right and left ear are reported for each patient. (NA=not available)

		Right ear						Left ear					
**Patient**	**Classification**	**250 Hz**	**500 Hz**	**1 kHz**	**2 kHz**	**4 kHz**	**8 kHz**	**250 Hz**	**500 Hz**	**1 kHz**	**2 kHz**	**4 kHz**	**8 kHz**
ID1	M2	100 dB	115 dB	115 dB	125 dB	125 dB	125 dB	80 dB	100 dB	95 dB	105 dB	110 dB	125 dB
ID2	M2	120 dB	120 dB	120 dB	120 dB	120 dB	120 dB	115 dB	125 dB	125 dB	125 dB	125 dB	125 dB
ID12	M2	40 dB	45 dB	45 dB	65 dB	90 dB	110 dB	55 dB	70 dB	80 dB	100 dB	120 dB	120 dB
ID19	M2	120 dB	120 dB	120 dB	120 dB	120 dB	120 dB	110 dB	110 dB	110 dB	110 dB	110 dB	110 dB
ID22	M2	35 dB	40 dB	65 dB	65 dB	100 dB	120 dB	65 dB	70 dB	80 dB	70 dB	80 dB	100 dB
ID7	M1	50 dB	55 dB	50 dB	50 dB	55 dB	60 dB	50 dB	50 dB	50 dB	40 dB	45 dB	40 dB
ID10	M1	30 dB	30 dB	50 dB	70 dB	70 dB	100 dB	122 dB	122 dB	122 dB	122 dB	122 dB	122 dB
ID14	M1	125 dB	125 dB	125 dB	125 dB	125 dB	125 dB	125 dB	125 dB	125 dB	125 dB	125 dB	125 dB
ID17	M1	35 dB	50 dB	50 dB	60 dB	50 dB	60 dB	45 dB	60 dB	70 dB	90 dB	100 dB	120 dB
ID18	M1	80 dB	95 dB	95 dB	105 dB	105 dB	120 dB	85 dB	90 dB	90 dB	100 dB	110 dB	120 dB
ID3	M0	30 dB	35 dB	35 dB	55 dB	50 dB	60 dB	100 dB	120 dB	120 dB	120 dB	120 dB	120 dB
ID4	M0	30 dB	45 dB	40 dB	40 dB	35 dB	20 dB	30 dB	45 dB	40 dB	40 dB	35 dB	20 dB
ID5	M0	45 dB	45 dB	50 dB	55 dB	40 dB	60 dB	75 dB	70 dB	75 dB	65 dB	120 dB	120 dB
ID6	M0	45 dB	45 dB	20 dB	15 dB	20 dB	35 dB	55 dB	50 dB	60 dB	80 dB	75 dB	105 dB
ID8	M0	30 dB	25 dB	20 dB	30 dB	100 dB	105 dB	25 dB	25 dB	20 dB	50 dB	95 dB	105 dB
ID9	M0	40 dB	50 dB	40 dB	30 dB	25 dB	25 dB	90 dB	90 dB	85 dB	70 dB	75 dB	75 dB
ID11	M0	55 dB	70 dB	105 dB	105 dB	110 dB	120 dB	55 dB	60 dB	60 dB	60 dB	50 dB	55 dB
ID13	M0	80 dB	75 dB	70 dB	75 dB	75 dB	70 dB	75 dB	85 dB	95 dB	90 dB	100 dB	120 dB
ID15	M0	70 dB	75 dB	90 dB	85 dB	80 dB	120 dB	35 dB	45 dB	45 dB	35 dB	30 dB	50 dB
ID16	M0	60 dB	65 dB	60 dB	70 dB	60 dB	60 dB	55 dB	65 dB	65 dB	70 dB	65 dB	60 dB
ID20	M0	25 dB	30 dB	40 dB	60 dB	75 dB	80 dB	25 dB	25 dB	25 dB	20 dB	55 dB	55 dB
ID21	M0	50 dB	40 dB	35 dB	65 dB	85 dB	105 dB	30 dB	30 dB	20 dB	25 dB	55 dB	90 dB
ID23	M0	75 dB	75 dB	90 dB	85 dB	90 dB	NA	80 dB	90 dB	95 dB	95 dB	95 dB	NA
ID24	M0	85 dB	90 dB	95 dB	95 dB	85 dB	120 dB	90 dB	100 dB	110 dB	120 dB	120 dB	120 dB

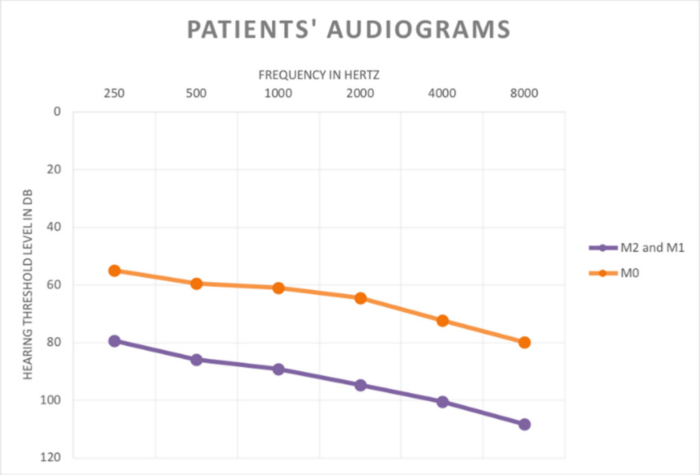

Figure S1: Mean audiometric pattern of carriers and non-carriers of pathogenic alleles. Comparison of the hearing threshold mean values at different frequencies between carriers of *SLC26A4* mutation (M2 and M1, purple line) and non carriers (orange line).
